# Noninvasive Assessment of β‐Secretase Activity Through Click Chemistry‐Mediated Enrichment of Neuronal Extracellular Vesicles to Detect Alzheimer's Disease

**DOI:** 10.1002/advs.202415289

**Published:** 2025-04-17

**Authors:** Hyoyong Kim, Junseok Lee, Audrey Qian, You‐Ren Ji, Ryan Zhang, Qixin Hu, Christopher Kazu Williams, Han‐Yu Chuang, Matthew D. Smalley, Yaya Xu, Liang Gao, Mary C. Mayo, Ting Zhang, Edwin M. Posadas, Zaldy S. Tan, Harry V. Vinters, Keith Vossel, Shino Magaki, Yazhen Zhu, Hsian‐Rong Tseng

**Affiliations:** ^1^ California NanoSystems Institute Crump Institute for Molecular Imaging Department of Molecular and Medical Pharmacology University of California, Los Angeles (UCLA) Los Angeles CA 90095 USA; ^2^ Department of Pathology and Laboratory Medicine David Geffen School of Medicine at UCLA Los Angeles CA 90095 USA; ^3^ Eximius Diagnostics Corp Magnify Incubator University of California Los Angeles (UCLA) Los Angeles CA 90095 USA; ^4^ Department of Neurology David Geffen School of Medicine at UCLA Los Angeles CA 90095 USA; ^5^ Division of Medical Oncology Department of Medicine Cedars‐Sinai Medical Center Los Angeles CA 90048 USA; ^6^ Departments of Neurology and Medicine Cedars‐Sinai Medical Center Los Angeles CA 90048 USA

**Keywords:** Alzheimer's disease, liquid biopsy, neurodegenerative disease, neuronal extracellular vesicle, β‐secretase

## Abstract

Alzheimer's disease (AD), the most prevalent type of dementia, is characterized by a biological process that begins with the development of AD neuropathologic change (ADNPC) while individuals remain asymptomatic. A key molecular hallmark of ADNPC is the accumulation of amyloid‐β plaques. β‐secretase plays a critical role in the upstream pathological cleavage of amyloid precursor protein (APP), producing amyloid‐β peptides that are prone to misfolding, ultimately contributing to plaque formation. Neuronal extracellular vesicles (NEVs) in the blood transport β‐secretase and preserve its activity, allowing for noninvasive profiling of β‐secretase activity for detecting early onset of ADNPC. In this study, a novel approach is approached for noninvasive assessment of β‐secretase activity in AD patients using an NEV β‐secretase activity assay. This assay identifies NEVs exhibiting colocalization of NEV markers with AD‐associated β‐secretase, generating a β‐secretase activity profile for each patient. The NEV β‐secretase activity assay represents a significant advancement in leveraging the diagnostic potential of NEVs, offering a noninvasive, quantitative method for reliably assessing β‐secretase activity to detect the early onset of ADNPC.

## Graphical abstract

This study presents the NEV β‐secretase activity assay, a groundbreaking method for noninvasive evaluation of β‐secretase activity in Alzheimer's disease (AD) patients, enabling the generation of individualized β‐secretase activity profiles. This quantitative and noninvasive approach provides a reliable means to assess β‐secretase activity, facilitating the early detection of AD neuropathologic changes (ADNPCs).

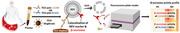

## 1 Introduction

Alzheimer's disease (AD), the most prevalent type of dementia in older adults,^[^ ^1^ ^]^ is characterized by a biological process that begins with AD neuropathologic changes (ADNPC).^[^ ^2^ ^]^ The accumulation of amyloid‐β plaques is a molecular hallmark of AD,^[^ ^3^ ^]^ occurring earlier than other ADNPCs, such as Tau‐mediated neuronal injury,^[^ ^4^ ^]^ and playing a critical role in disrupting neuronal function and contributing to neurodegeneration. In response, immunotherapies using monoclonal antibodies have been recently developed to target and clear brain amyloid in early‐stage AD patients.^[^ ^5^ ^]^ While these treatments have effectively reduced amyloid burden, the corresponding improvements in cognition and function have been modest, and the disease has continued to progress even after amyloid clearance.^[^ ^6^ ^]^ Therefore, despite these therapeutic advances, earlier detection of ADNPC before significant amyloid‐β accumulation has become crucial for timely intervention and early disease treatment.

**Figure 1:**
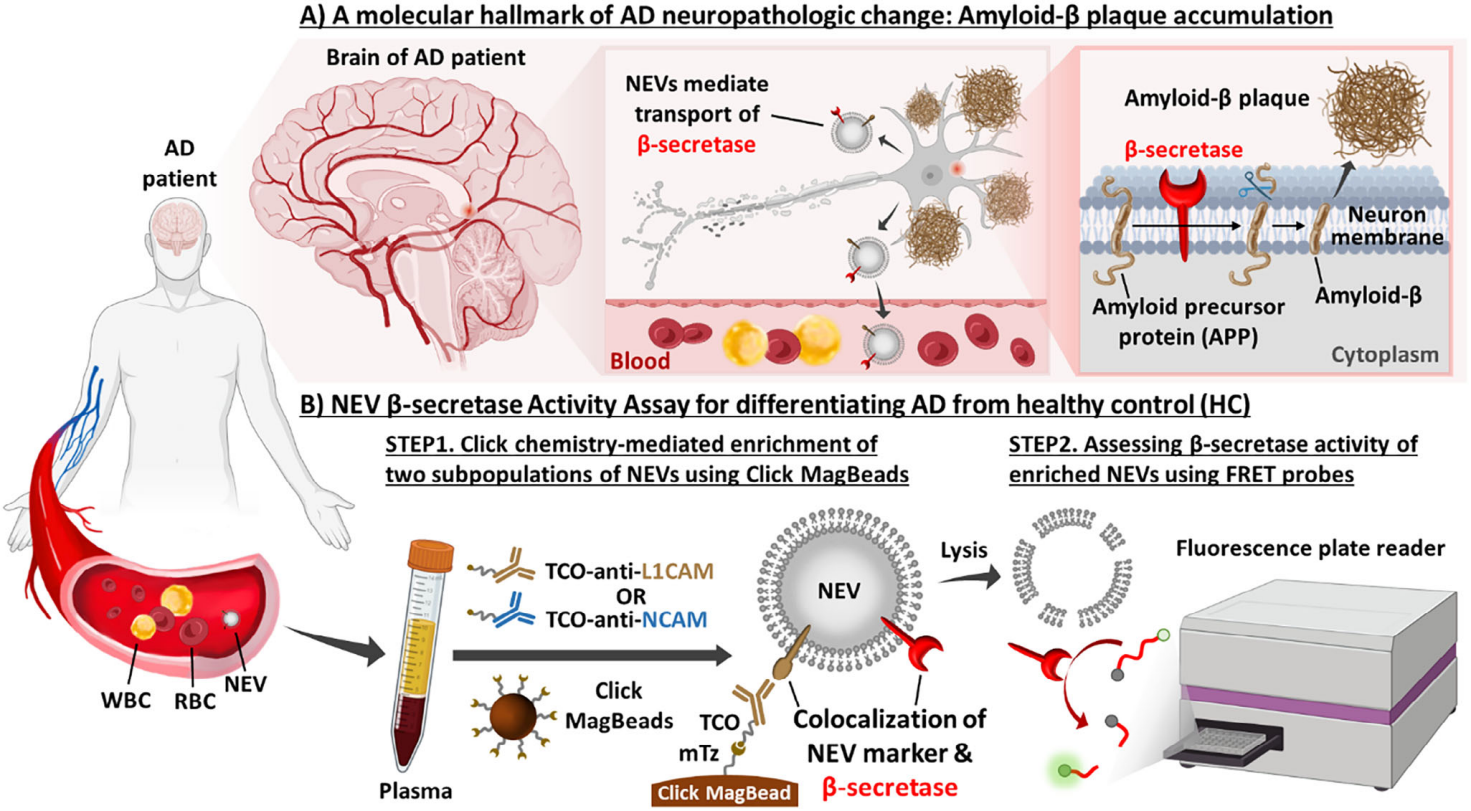
A) A molecular hallmark of ADNPC: Amyloid‐β plaque accumulation. β‐secretase drives the formation of amyloid‐β plaques, a molecular hallmark of ADNPC, by initiating an abnormal cleavage of APP on the neuron membrane. NEVs mediate the transport of β‐secretase from their parental neurons, while retaining its functional activity. Given these features, NEVs serve as ideal blood‐based biomarkers, offering a noninvasive approach to assess β‐secretase activity for detecting the early onset of ADNPC. B) NEV β‐secretase activity assay for differentiating AD from HC. Step 1. Click chemistry‐mediated enrichment of two subpopulations of NEVs using Click MagBeads. After labeling NEVs in 0.4 mL of plasma by one of the two TCO‐grafted antibodies targeting NEV markers (i.e., L1CAM or NCAM), Click MagBeads functionalized with methyltetrazine (mTz) were employed to immobilize the respective subpopulations of NEVs. Step 2. Assessment of β‐secretase activity of enriched NEVs using FRET probes. To assess β‐secretase activity, NEVs enriched on Click MagBeads were lysed and incubated with β‐secretase FRET probes. The central premise is that positive readouts are generated only when NEVs exhibit colocalization of one of the two NEV markers (i.e., L1CAM or NCAM) with β‐secretase. The resulting β‐secretase activity profiles across two subpopulations of NEVs, L1CAM(+)‐NEVs and NCAM(+)‐NEVs, were employed to differentiate AD patients from HCs.

Under normal physiological conditions, α‐secretase predominantly cleaves the amyloid‐β domain within the amyloid precursor protein (APP), producing short, soluble peptide fragments. Conversely, in the pathological pathway, β‐secretase (e.g., beta‐site APP cleaving enzyme 1, BACE1) competes with α‐secretase for APP cleavage, producing amyloid‐β peptides (e.g., amyloid‐β 42).^[^ ^7^ ^]^ These peptides are prone to misfolding, ultimately leading to the formation of amyloid‐β plaques (**Figure** **1**A).^[^ ^3a^ ^]^ Current diagnostic methods, such as cerebrospinal fluid (CSF) tests and positron emission tomography (PET) scans,^[^ ^8^ ^]^ can only detect amyloid plaque burden after amyloid‐β aggregates have accumulated to detectible levels. Given that amyloid‐β accumulation is triggered by β‐secretase, detecting elevated β‐secretase activity could offer a promising alternative approach for identifying the onset of ADNPC at even earlier stages (Figure S1, Supporting Information).^[^ ^9^ ^]^

Previous studies have demonstrated significantly elevated β‐secretase activity in AD patients’ brain tissues.^[^ ^10^ ^]^ However, assessing β‐secretase activity in brain tissue is limited to postmortem analysis due to the highly invasive nature of tissue collection. CSF has also been explored as an alternative for assessing β‐secretase activity.^[^ ^11^ ^]^ However, the collection of CSF via lumbar puncture remains invasive and carries risks, making it impractical for routine surveillance that requires repeated sampling.^[^ ^12^ ^]^ To overcome this challenge, researchers have investigated the assessment of β‐secretase activity in plasma and serum samples from AD patients.^[^ ^13^ ^]^ While elevated enzyme activity has been detected, the ability to distinguish AD patients from healthy controls (HCs) remains limited, likely because β‐secretase in blood can originate from multiple organs. Developing a method to selectively assess β‐secretase activity from neuronal sources could significantly improve its diagnostic performance, particularly for detecting early onset of ADNPC.

As a key component of liquid biopsies, extracellular vesicles (EVs) are promising candidates as noninvasive biomarkers for disease diagnosis and surveillance.^[^ ^14^ ^]^ These heterogeneous, nano‐scaled particles, enclosed by phospholipid bilayers, are released by various cell types, including neurons.^[^ ^15^ ^]^ EVs protect fragile biomolecular cargos, such as proteins, RNA, and DNA, thereby reflecting the biomolecular profile of the parental cells or tissues throughout disease progression.^[^ ^16^ ^]^ Neuronal EVs (NEVs) are particularly suited for noninvasive harvesting of β‐secretase of neuronal origin due to three key advantages: i) NEVs mediate the transport of biomolecular cargos, including β‐secretase, from neurons across the blood‐brain barrier into the bloodstream,^[^ ^17^ ^]^ ii) NEV membranes protect fragile biomolecules, thus preserving the integrity and activity of β‐secretase;^[^ ^18^ ^]^ and iii) NEVs mirror the surface markers of their parental neurons, allowing the use of preestablished neuron surface markers for NEV isolation.^[^ ^19^ ^]^ These features permit the assessment of β‐secretase activity within NEVs from blood samples, providing an opportunity for detecting the earlier onset of ADNPC in a noninvasive manner.

To harness NEVs’ potential for assessing β‐secretase activity in the brain, it is crucial to develop a system that enriches NEVs while preserving enzyme activity. Conventional methods relying on immunoaffinity‐based approaches targeting NEV markers often require antibody precoating through techniques such as amide coupling or biotin‐streptavidin interaction.^[^ ^20^ ^]^ These methods can present challenges in capture efficiency and potential biological interference. To address these challenges, our team pioneered two innovative click chemistry‐mediated EV enrichment technologies, namely Click Chips and Click Beads.^[^ ^21^ ^]^ These technologies offer two key advantages: i) click motif‐grafted antibodies used to label EVs significantly reduce antibody consumption (by 10–100 times) and labeling time, and ii) the use of an inverse electron demand Diels‐Alder reaction between trans‐cyclooctene (TCO) and tetrazine (Tz) motifs ensures a bioorthogonal click reaction,^[^ ^22^ ^]^ circumventing the biological interferences commonly encountered with biotin‐streptavidin interaction. Additionally, our past studies have demonstrated that click chemistry‐mediated EV enrichment can be seamlessly coupled with various downstream molecular analyses, such as mRNA profiling and protein detection.^[^ ^21^, ^23^ ^]^

Here, we introduce an NEV β‐secretase activity assay for the noninvasive detection of early onset of ADNPC. As illustrated in Figure 1B, this assay is comprised of two main steps: i) click chemistry‐mediated enrichment of two subpopulations of NEVs using Click MagBeads with one of the two TCO‐grafted antibodies targeting NEV markers, i.e., L1 cell adhesion molecule (L1CAM) and neural cell adhesion molecule (NCAM), and ii) assessment of β‐secretase activity of the enriched NEVs using β‐secretase FRET probes, providing a β‐secretase activity profile for each AD patient. The core concept of this technology is that NEVs exhibiting colocalization of one of the two NEV markers (i.e., L1CAM or NCAM) with β‐secretase can exclusively generate positive readouts. This assay demonstrated excellent diagnostic accuracy in differentiating AD patients from HCs, achieving an area under receiver operating characteristic curve (AUROC) of 0.986. Importantly, the established scoring system showed a strong correlation with the AD patients’ cognitive performance indicated by the Mini‐Mental State Examination (MMSE) score.

## 2 Results

### 2.1 Identification of NEV Markers

Since the NEV β‐secretase activity assay is designed to specifically detect NEVs which exhibit colocalization of NEV markers with β‐secretase (Figure 1B), we first conducted a comprehensive literature survey to identify appropriate NEV markers for NEV enrichment. For NEV marker selection, we analyzed a recent systematic review that focused on the enrichment of NEVs from the blood of neurodegenerative disease patients.^[^ ^24^ ^]^ This review evaluated publications over the past decade, with a primary emphasis on NEV enrichment from blood via immunoprecipitation, and identified L1CAM as the most commonly used marker for isolating NEVs. Despite ongoing debates regarding reliability of L1CAM as a marker for NEV isolation,^[^ ^25^ ^]^ its widespread application as a primary marker in the NEV research field justified incorporating this maker to our assay. Additionally, we sought to identify complementary markers to enhance assay performance. As outlined in Table S1 (Supporting Information), our literature analyses highlighted NCAM as another widely used marker that could complement L1CAM for enriching NEVs. Based on these findings, we incorporated both L1CAM and NCAM to improve the robustness and performance of the NEV β‐secretase activity assay. In parallel, previous studies have demonstrated that NEVs can transport key enzymes involved in the APP cleavage pathway, including β‐secretase.^[^ ^26^ ^]^ Additionally, research has indicated that β‐secretase expression level and activity in other neurodegenerative diseases, such as Parkinson's disease and frontotemporal dementia, are significantly lower than in AD.^[^ ^27^ ^]^ Leveraging these insights, we designed the NEV β‐secretase activity assay to assess β‐secretase activity from two subpopulations of NEVs: L1CAM(+)‐NEVs and NCAM(+)‐NEVs.

### 2.2 Characterization of NEVs in the CSF of AD Patient

As a model target to demonstrate our assay, NEVs were isolated from the CSF of AD patient via polyethylene glycol (PEG)‐based precipitation using ExoQuick (detailed in Experimental Section).^[^ ^28^ ^]^ The characterization of the isolated NEVs followed the guidelines established by the International Society for Extracellular Vesicles (MISEV2023).^[^ ^29^ ^]^ Nanoparticle tracking analysis (NTA) determined the size and concentration of NEVs as 171.5 ± 59 nm and 3.19 × 10^9^ NEVs mL^−1^, respectively (Figure S2A, Supporting Information). The NEVs’ morphology and structural integrity were further validated by transmission electron microscopy (TEM, Figure S2B, Supporting Information) and scanning electron microscopy (SEM, Figure S2C, Supporting Information).

### 2.3 Preparation and Characterization of Click MagBeads

**Figure 2:**
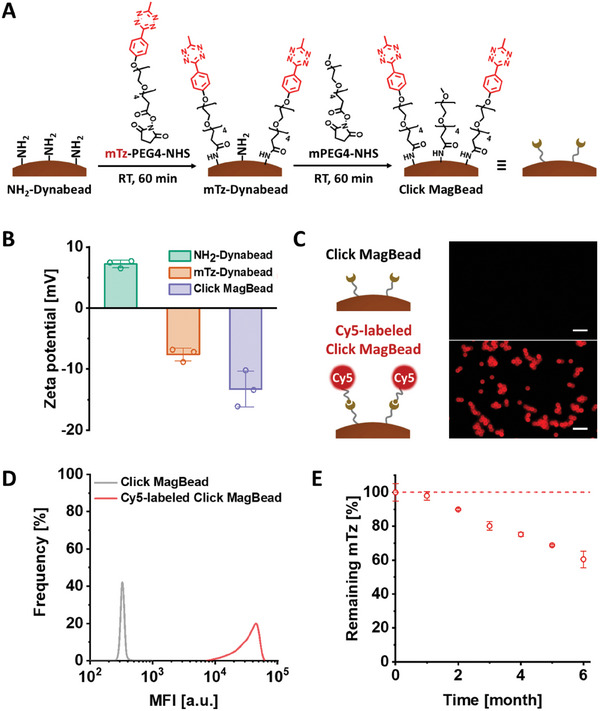
Preparation and characterization of Click MagBeads. A) Schematic illustration of stepwise preparation of the Click MagBeads. B) Zeta potentials of the amine‐grafted Dynabeads™ (NH_2_‐Dynabeads), mTz‐modified Dynabeads™ (mTz‐Dynabeads), and Click MagBeads. C) Fluorescence micrographs of the Click MagBeads before and after labeling with TCO‐Cy5 (scale bar = 10 µm) and D) corresponding histograms of mean fluorescence intensity (MFI). E) Measurement of mTz motif concentration on the Click MagBeads over time after synthesis.

Building on our previous development of Click Beads,^[^ ^21c,d^ ^]^ a click chemistry‐mediated EV enrichment technology, we have developed the Click MagBeads platform, introducing two major enhancements: i) the transition from silica beads to magnetic beads, offering key advantages for EV enrichment including ease of separation, lower risk of sample loss, greater scalability, and enhanced reproducibility,^[^ ^30^ ^]^ and ii) the replacement of tetrazine (Tz) with methyltetrazine (mTz), enhancing stability of surface click motifs and extending their storage life from two weeks to over three months. As outlined in **Figure** **2**A, the synthesis of Click MagBeads follows a two‐step process, adapted from the synthetic procedure of our silica‐based Click Beads.^[^ ^21c,d^ ^]^ We modified amine‐grafted Dynabeads (2.7 µm diameter, ThermoFisher) by conjugating with methyltetrazine‐PEG4‐*N*‐hydroxysuccinimide (mTz‐PEG4‐NHS) ester via NHS ester chemistry to produce mTz‐Dynabeads. Any residual amine groups on the Dynabeads, inevitably remaining even after complete functionalization, were subsequently passivated with methoxypolyethylene glycol succinate‐*N*‐hydroxysuccinimide (mPEG4‐NHS) ester to confer anti‐fouling properties,^[^ ^31^ ^]^ yielding the final Click MagBeads.

We monitored the changes in the surface chemistry of the magnetic beads by measuring zeta potential shifts (Figure 2B). The amine‐grafted Dynabeads initially exhibited a strong positive charge, which gradually decreased following the sequential addition of mTz‐PEG4‐NHS and mPEG4‐NHS, confirming successful attachment of both functional groups. The presence of mTz motifs on the Click MagBeads was further validated by a click reaction with TCO‐labeled Cy5 (TCO‐Cy5), resulting in noticeable Cy5 fluorescence under fluorescence microscopy (Figure 2C). A corresponding histogram of mean fluorescence intensity (MFI) also confirmed the successful interaction between mTz and TCO motifs (Figure 2D).

To measure the density of mTz motifs on the Click MagBeads and assess their stability over time, we employed the TCO‐Cy5 labeling method (Figure 2E, see details in Experimental Section). The initial mTz motif concentration was 0.464 ± 0.024 nmol mg^−1^, with approximately 60% retention after six months. Based on this result, all experiments were conducted within two months of bead synthesis, ensuring that over 80% of the mTz motifs remained active. Notably, the quantity of retained mTz motifs on the Click MagBeads (over 80% retention, 9 pmol per batch) was sufficient to capture all TCO‐grafted antibodies used in this study (100 ng antibody, equivalent to 2 pmol TCO motifs per batch).

### 2.4 Verification of Click Chemistry‐Mediated NEV Enrichment

**Figure 3:**
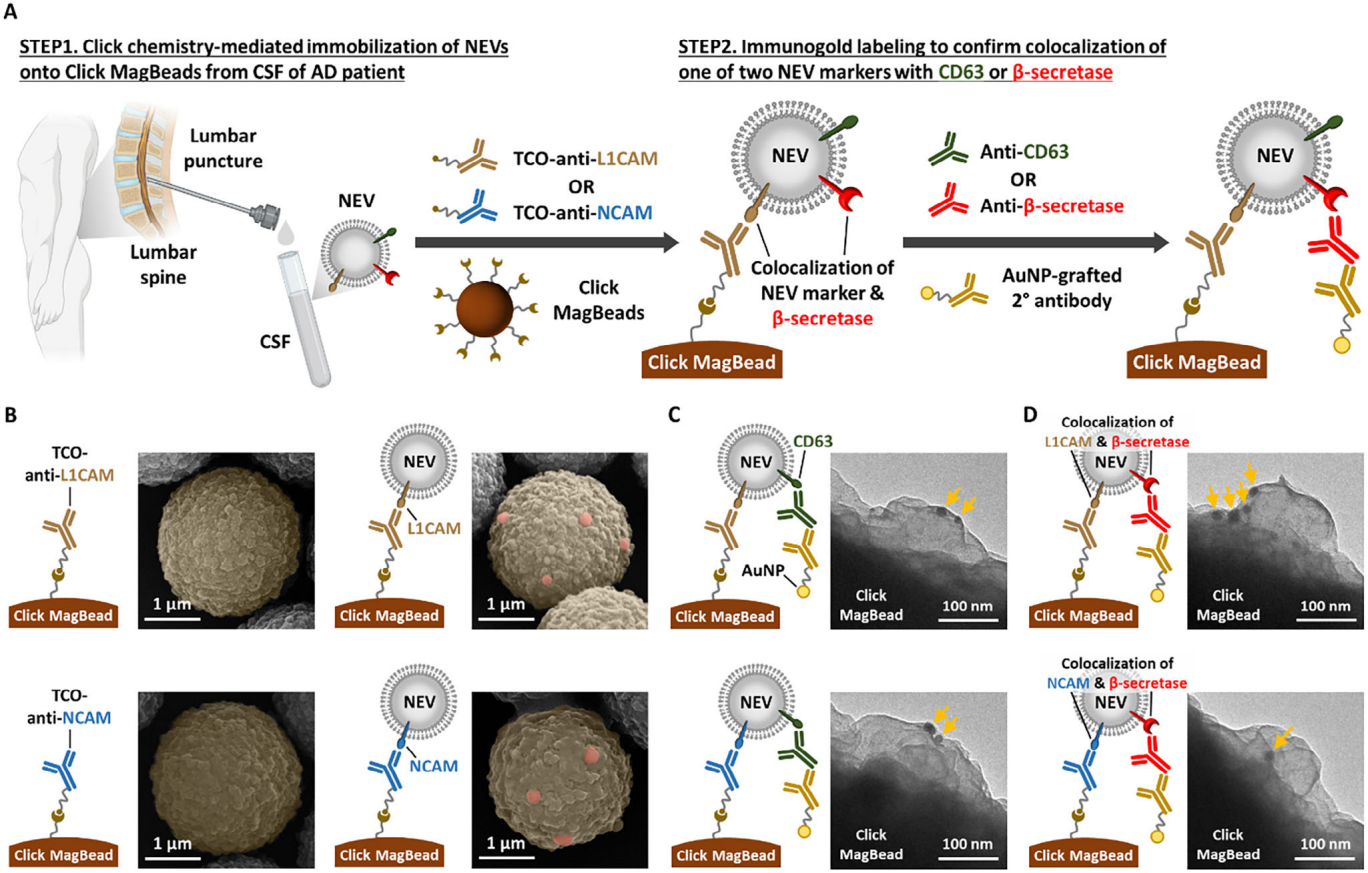
Verification of click chemistry‐mediated enrichment of two subpopulations of NEVs and confirmation of colocalization of NEV markers with β‐secretase on the enriched NEVs. A) A schematic illustration of the workflow for characterizing interface between NEVs and Click MagBeads. Step 1. Click chemistry‐mediated immobilization of NEVs onto Click MagBeads from CSF of AD patient. NEVs in 0.4 mL CSF of AD patient were labeled with one of the two TCO‐grafted antibodies targeting NEV markers (i.e., L1CAM or NCAM). Click MagBeads were then employed to immobilize the respective subpopulations of NEVs. Step 2. Immunogold labeling to confirm colocalization of one of the two NEV markers with CD63 or β‐secretase. To verify the colocalization of one of the two NEV markers with CD63, a well‐established EV marker, or β‐secretase, enriched NEVs were labeled with one of the two antibodies targeting CD63 or β‐secretase, followed by immunogold labeling using AuNP‐grafted secondary antibody. B) SEM images of Click MagBeads without (left) and with (right) the immobilized NEVs using TCO‐anti‐L1CAM (top) and TCO‐anti‐NCAM (bottom). C,D) TEM images of L1CAM(+)‐NEVs (top) and NCAM(+)‐NEVs (bottom) immobilized onto the Click MagBeads with immunogold labeling of C) CD63 and D) β‐secretase.

To verify the click chemistry‐mediated enrichment of two subpopulations of NEVs, we implemented the workflow illustrated in STEP 1 of **Figure** **3**A. Specifically, 0.4 mL of CSF, collected from AD patient, was incubated with 100 ng of one of the two TCO‐grafted antibodies targeting NEV markers, i.e., TCO‐anti‐L1CAM or TCO‐anti‐NCAM. The labeled NEVs were subsequently enriched onto the Click MagBeads through the click chemistry interaction between mTz and TCO motifs, as shown in the insets of Figure 3B. SEM images in Figure 3B provided direct visualization of the two subpopulations of NEVs enriched onto the Click MagBeads, with their size and morphology aligning with those of intact NEVs (Figure S2, Supporting Information).

To further confirm the identity of the enriched particles, immunogold staining was performed, as outlined in STEP 2 of Figure 3A. NEVs enriched onto the Click MagBeads were labeled with an antibody against CD63, a well‐established EV marker, followed by tagging with a gold nanoparticle (AuNP)‐grafted secondary antibody, as depicted in the insets of Figure 3C. TEM images in Figure 3C validated the presence of AuNPs on both NEV subpopulations enriched onto the Click MagBeads, confirming the successful application of our Click MagBeads platform for click chemistry‐mediated NEV enrichment.

### 2.5 Confirmation of NEV Marker/β‐Secretase Colocalization

With the designed working mechanism of the NEV β‐secretase activity assay (Figure 1B), positive readouts are generated only when NEVs exhibit colocalization of one of the two NEV markers (i.e., L1CAM or NCAM) with β‐secretase. To confirm this colocalization, we performed the immunogold staining by labeling the enriched NEVs with anti‐β‐secretase antibody, followed by the application of AuNP‐grafted secondary antibody, as outlined in STEP 2 of Figure 3A. This approach allowed us to label the AuNPs onto the NEV‐associated β‐secretase, as illustrated in the insets of Figure 3D. The TEM images in Figure 3D confirmed the presence of β‐secretase on both NEV subpopulations, demonstrating the colocalization of NEV markers with β‐secretase on NEVs enriched onto the Click MagBeads. This finding highlights the potential for assessing β‐secretase activity from the enriched NEVs. Furthermore, these results establish a solid foundation for the targeted assessment of β‐secretase activity from specific NEV subpopulations in complex biological fluids, such as blood.

### 2.6 Investigation of β‐Secretase Enzymatic Kinetics

After confirming the colocalization of NEV markers with β‐secretase on the enriched NEVs, we proceeded to optimize the assessment of β‐secretase activity using recombinant human β‐secretase and β‐secretase FRET probe (Figure S3A, Supporting Information, see details in Experimental Section). An enzyme kinetics study was performed to establish an optimal substrate concentration to operate the NEV β‐secretase activity assay. Specifically, we measured the hydrolysis rate of β‐secretase FRET probes at various concentrations with a fixed amount of β‐secretase (Figure S3B, Supporting Information). The resulting data were fitted to the Michaelis‐Menten equation to determine the kinetic parameters of β‐secretase, yielding a Michaelis constant (K_M_) of 3.8 µM (Figure S3C, Supporting Information), consistent with previously reported values.^[^ ^32^ ^]^ Based on this result, we selected the FRET probe concentration of 12 µM, approximately three times the K_M_, ensuring at least 75% of the maximum reaction rate (V_max_ = 2.512 × 10^−3^ µM s^−1^) during the assay. We then assessed the dynamic range of β‐secretase activity across a concentration range of 1.8–160 nM, with a detection limit of 1.6 nM (Figure S3D, Supporting Information). Furthermore, we evaluated the specificity of the β‐secretase FRET probe against non‐target proteases, revealing that the activities from non‐target proteases were less than 6% compared to β‐secretase (Figure S3E, Supporting Information). These results confirmed the high specificity of the β‐secretase FRET probe utilized in this study.

### 2.7 Proof‐of‐Concept Study with Clinical CSF Sample

**Figure 4:**
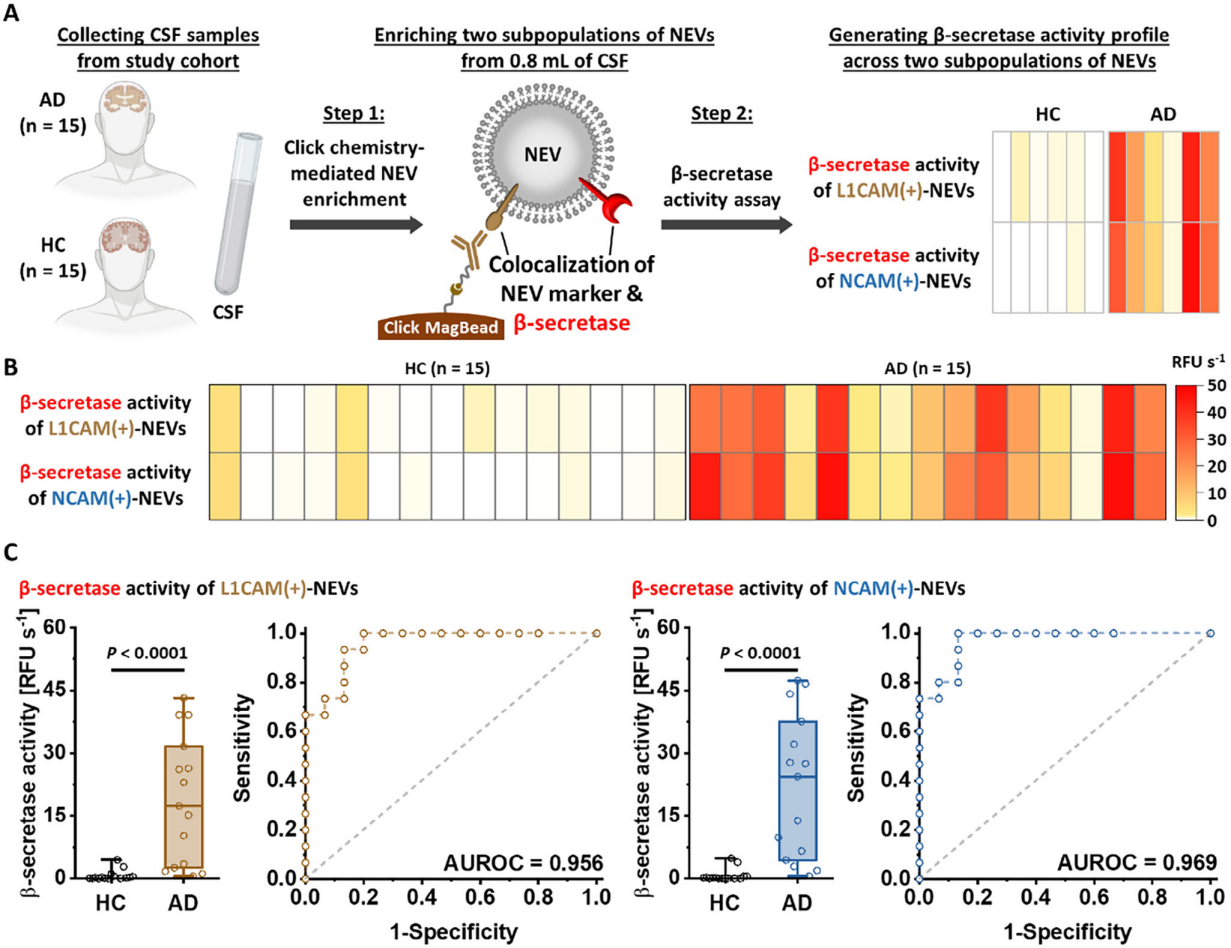
Proof‐of‐concept study of the NEV β‐secretase activity assay with clinical CSF samples in a study cohort. A) A general workflow adopted for the NEV β‐secretase activity assay using clinical CSF samples, each with a volume of 0.8 mL, from a study cohort of 15 AD patients and 15 HCs. The two subpopulations of NEVs from the CSF samples were enriched onto the Click MagBeads using one of the two TCO‐grafted antibodies targeting NEV markers, i.e., L1CAM and NCAM. To assess β‐secretase activity, the enriched NEVs were lysed and incubated with a β‐secretase FRET probe to generate the β‐secretase activity profile of study cohort. B) Heatmap summarizing quantitative β‐secretase activity readouts obtained from the two subpopulations of NEVs in the clinical CSF samples. C) Box charts and corresponding ROC curves of two subpopulations of NEVs for differentiating AD patients from HCs.

Before testing plasma samples, we performed a proof‐of‐concept demonstration of the NEV β‐secretase activity assay using clinical CSF samples, following the workflow shown in **Figure** **4**A. NEVs were enriched from 0.8 mL of clinical CSF samples (0.4 mL for each NEV subpopulation), collected from a study cohort of 15 AD patients and 15 HCs, onto the Click MagBeads (see procedures described in 2.4. Verification of Click Chemistry‐Mediated NEV Enrichment section and details in Experimental Section). The clinical characteristics of these participants are summarized in Table S2 (Supporting Information). Subsequently, the enriched NEVs underwent lysis to release β‐secretase, followed by incubation with β‐secretase FRET probes to measure enzyme activity. By assessing β‐secretase activity in two subpopulations of NEVs, we generated a β‐secretase activity profile for each patient. The quantitative β‐secretase activity data were organized into a heatmap (Figure 4B), showing significantly higher β‐secretase activity in AD patients compared to HCs, with p‐values less than 0.0001 for both L1CAM(+)‐NEVs and NCAM(+)‐NEVs (Figure 4C). The diagnostic performance was further evaluated using AUROC values, yielding 0.956 and 0.969 for L1CAM(+)‐NEVs and NCAM(+)‐NEVs, respectively. This study successfully demonstrated the capability of the NEV β‐secretase activity assay to reliably differentiate AD patients from HCs by assessing β‐secretase activity of NEVs.

### 2.8 NEV β‐Secretase Activity Assay with Synthetic Plasma Sample

**Figure 5:**
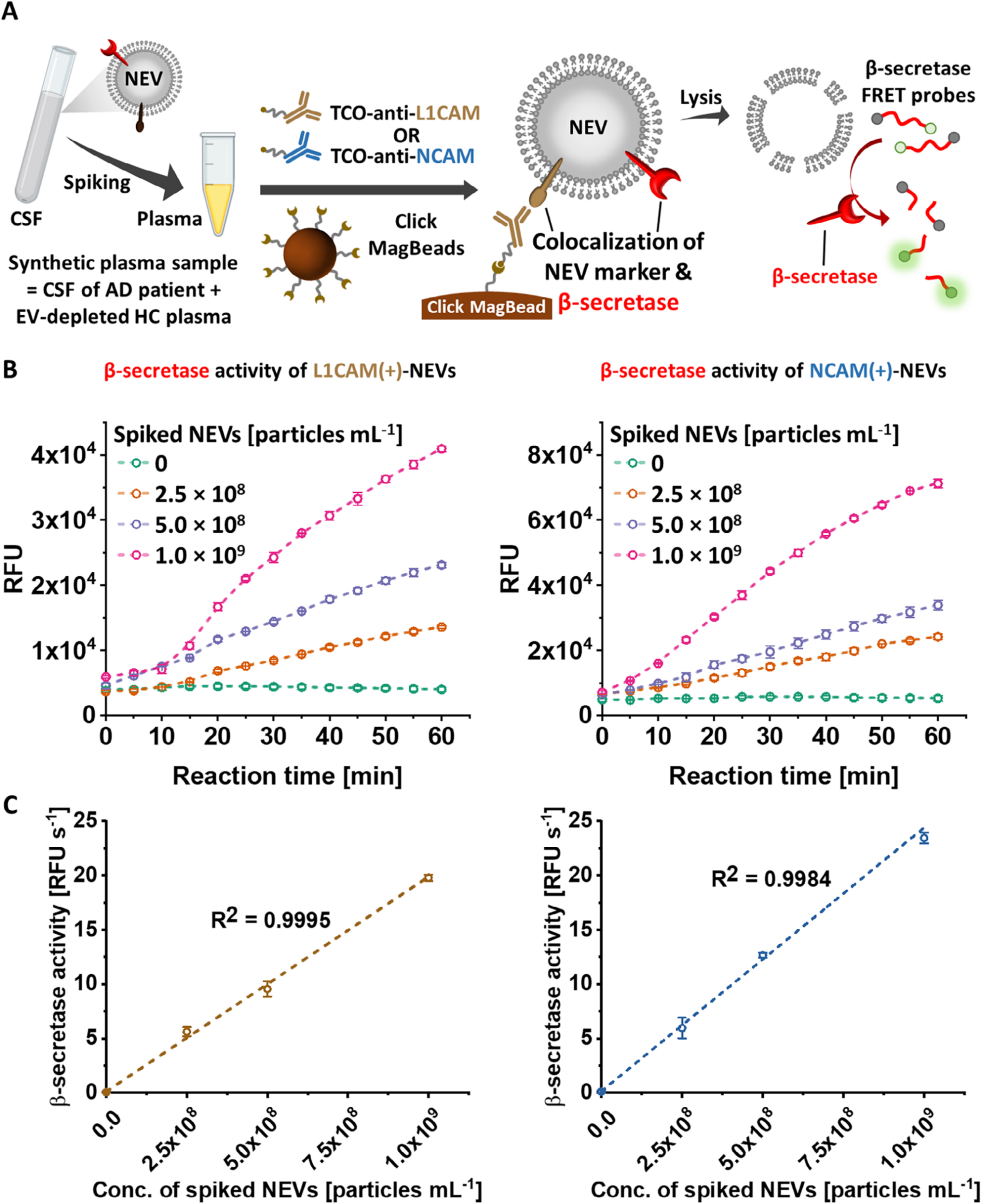
Validation of the NEV β‐secretase activity assay with synthetic plasma samples. A) A schematic illustration of the workflow developed for assessing the capability of the NEV β‐secretase activity assay to detect NEVs in synthetic plasma samples. Synthetic plasma samples were prepared by spiking the CSF of AD patient into EV‐depleted HC plasma. B) Time‐dependent measurement of fluorescence generated by the hydrolysis of the β‐secretase FRET probes by β‐secretase of two subpopulations of NEVs enriched with TCO‐anti‐L1CAM (left) and TCO‐anti‐NCAM (right) across varying concentrations of spiked NEVs. C) Corresponding calibration curves of β‐secretase activity of two subpopulations of NEVs across varying concentrations of spiked NEVs.

Following the successful proof‐of‐concept study of the NEV β‐secretase activity assay using clinical CSF samples, we further validated its reliability for detecting NEVs in plasma, a more complex biological matrix than CSF, by conducting experiments with synthetic plasma samples. As shown in **Figure** **5**A, we prepared synthetic plasma samples by spiking the CSF of AD patient into EV‐depleted HC plasma. The concentration of NEVs in the CSF of AD patient was determined by NTA (see details in Experimental Section). The prepared synthetic plasma samples were then divided into duplicates, each containing 0.4 mL, to assess β‐secretase activity of each NEV subpopulation by following the same procedure employed in the CSF sample assay. The results of time‐dependent measurement presented in Figure 5B proved gradual fluorescence signal enhancement in accordance with the increase of the concentration of spiked NEVs, from 0 to 10^9^ NEVs mL^−1^, for both NEV subpopulations. Additionally, we established the calibration curves of β‐secretase activity of two subpopulations of NEVs across varying concentrations of spiked NEVs (Figure 5C). The results demonstrated a strong linear correlation (R^2^ > 0.99) of β‐secretase activity in both NEV subpopulations across a wide concentration range of spiked NEVs, with a dynamic range of 5 × 10^7^–10^9^ NEVs mL^−1^. This outcome validated the assay's ability to quantitatively detect NEVs even in plasma, ensuring its applicability for quantifying NEV‐associated β‐secretase in plasma and holding promise for broader clinical applications in noninvasive ADNPC diagnostics.

### 2.9 Retrospective Case‐Control Study with Clinical Plasma Sample

**Figure 6:**
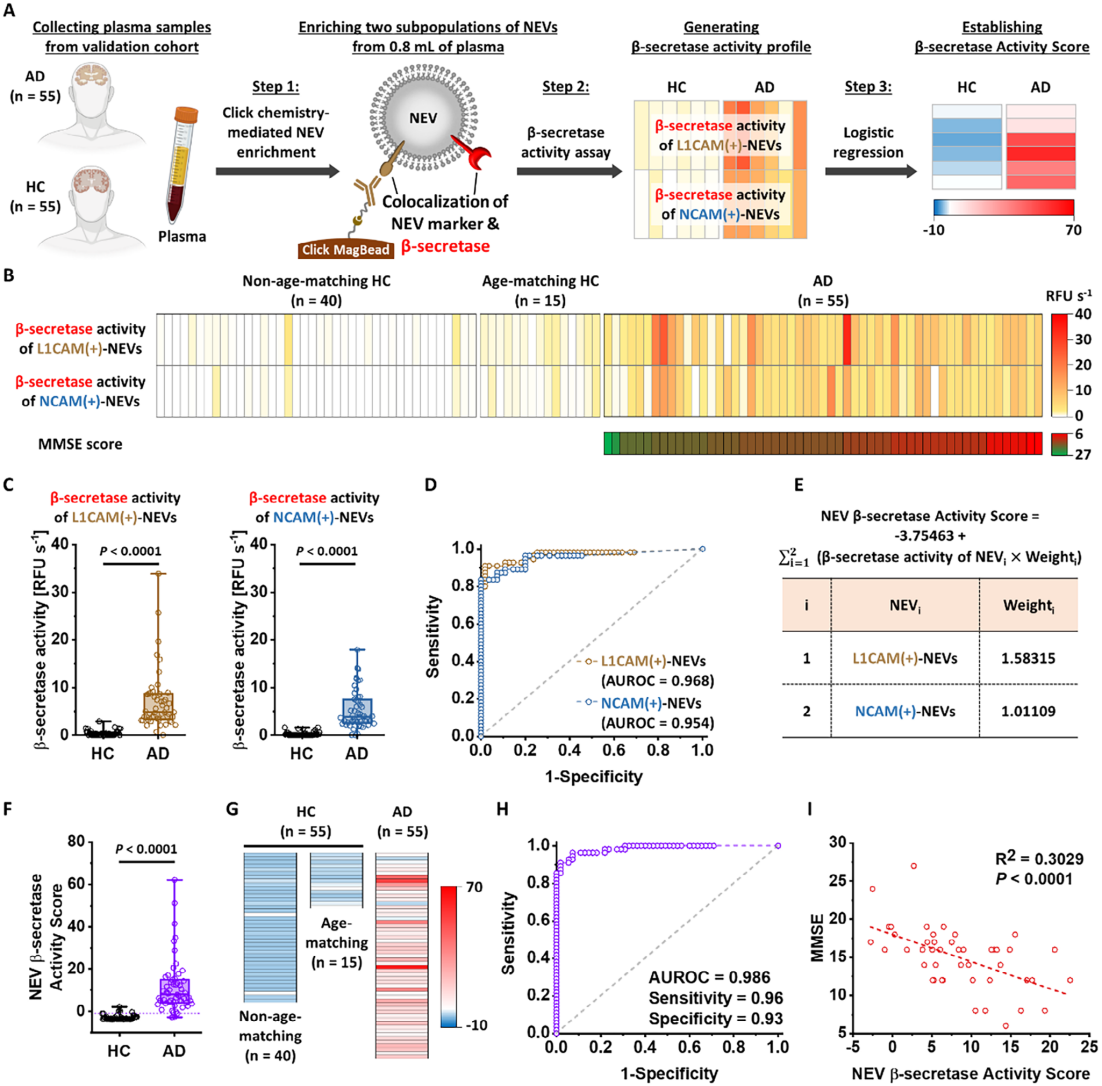
Retrospective case‐control study with clinical plasma samples in a validation cohort. A) A general workflow adopted for the NEV β‐secretase activity assay using clinical plasma samples from a validation cohort of 55 AD patients and 55 HCs. B) Heatmap summarizing quantitative β‐secretase activity readouts obtained from the two subpopulations of NEVs in the clinical plasma samples from the validation cohort and their corresponding MMSE scores. C) Box charts and D) corresponding ROC curves of β‐secretase activity readouts from two subpopulations of NEVs for differentiating AD patients from HCs. E) NEV β‐secretase Activity Score established based on a logistic regression model that synergistically combines the readouts from two subpopulations of NEVs into a single metric. F) Box chart, G) heatmap, and H) ROC curve of NEV β‐secretase Activity Score to demonstrate the enhanced differentiation of AD patients from HCs. I) Correlation between NEV β‐secretase Activity Scores and cognitive performance of AD patients indicated by MMSE scores.

Building on the successful validation of the NEV β‐secretase activity assay with synthetic plasma samples, we proceeded with a retrospective case‐control study with clinical plasma samples, following the workflow illustrated in **Figure** **6**A. We assessed β‐secretase activity of the two subpopulations of NEVs in the clinical plasma samples collected from the validation cohort of 55 AD patients and 55 HCs, as detailed in Table S3 (Supporting Information). The β‐secretase activity data, presented in a heatmap (Figure 6B), confirmed that the NEV β‐secretase activity assay can effectively distinguish AD patients from HCs by analyzing clinical plasma samples, with p‐values less than 0.0001 for both NEV subpopulations (Figure 6C). To thoroughly confirm that the observed differentiation of AD patients from HCs was attributed not to variations in β‐secretase expression levels but to its intrinsic enzymatic activity, we conducted an ELISA‐based quantification of β‐secretase expression levels within the enriched NEVs (Figure S4, Supporting Information). The results demonstrated that β‐secretase expression levels did not significantly differ between AD and HC groups, whereas β‐secretase activity assessments achieved clear differentiation, confirming that our assay specifically reflects enzymatic activity rather than merely quantifying protein abundance. Similar to the CSF sample study, the assay's diagnostic performance was further assessed using AUROC values, which were 0.968 for L1CAM(+)‐NEVs and 0.954 for NCAM(+)‐NEVs (Figure 6D). Notably, the diagnostic accuracy in differentiating AD patients from age‐matching HCs was comparable to that for non‐age‐matching HCs (Figure S5, Supporting Information). Additionally, we established a logistic regression model that synergistically combines the readouts from two subpopulations of NEVs into a single metric, termed the NEV β‐secretase Activity Score (Figure 6E). The NEV β‐secretase Activity Score demonstrated significant differentiation (*p* < 0.0001) of AD patients from HCs (Figure 6F,G) and exhibited enhanced discrimination power, achieving an AUROC of 0.986, with high sensitivity (96%) and specificity (93%) at the optimal cutoff value of −0.99 (Figure 6H). Importantly, our method achieved significantly superior diagnostic performance compared to previous non‐EV‐based β‐secretase detection approaches (Table S4, Supporting Information). We also confirmed that the NEV β‐secretase activity scores from the majority of AD patients were negatively correlated with their Mini‐Mental State Examination (MMSE) scores (Figure 6I) with R^2^ value of 0.3029. This correlation strength exceeds that of previously reported study for establishing other blood biomarkers for AD detection,^[^ ^33^ ^]^ reinforcing the relevance and clinical significance of our findings. Notably, in the same manner, we assessed the potential effect of age on our assay's readouts by correlating the ages of AD patients with their respective NEV β‐secretase Activity Scores. As shown in Figure S6 (Supporting Information), the analysis revealed no statistically significant correlation, demonstrating that our assay specifically reflects AD‐related β‐secretase activity rather than being influenced by age differences. These results underscore the capability of this assay to detect AD using clinical plasma samples, enabling a noninvasive assessment of the ADNPC.

## 3 Discussion

The ideal approach for managing AD is to detect the early onset of ADNPC, enabling timely intervention, early treatment, and slowing of disease progression. Current FDA‐approved diagnostic modalities, such as PET scans and CSF tests,^[^ ^8^ ^]^ primarily detect the accumulation of amyloid‐β plaques, which are the products of the pathological APP cleavage pathway and key early indicators of ADNPC. PET scans, for instance, utilize PET probes developed from amyloid‐binding dyes that insert into the β‐sheet structure of amyloid‐β plaques.^[^ ^34^ ^]^ As a more cost‐effective and accessible alternative, CSF samples are analyzed using immunoassays to measure the ratio of amyloid‐β 42 to amyloid‐β 40.^[^ ^8^, ^35^ ^]^ While these existing diagnostic modalities are valuable, they typically identify ADNPC after significant amyloid‐β accumulation has occurred in the brain. Since amyloid‐β accumulation is triggered by the β‐secretase,^[^ ^7^ ^]^ detecting elevated β‐secretase activity could offer an alternative approach for detecting even earlier onset of ADNPC (Figure S1, Supporting Information). To fulfill this purpose, we developed the NEV β‐secretase activity assay, a noninvasive blood test that assesses the activity of β‐secretase, offering the potential for earlier detection of ADNPC compared to existing methods and therefore paving the way for more timely intervention in AD progression.

To develop the NEV β‐secretase activity assay, we first conducted a literature review to identify the most reliable NEV markers for the specific enrichment of NEVs. Among the markers considered, L1CAM and NCAM emerged as the most well‐established NEV markers over the last decade.^[^ ^24^, ^25^, ^36^ ^]^ In parallel, we identified NEVs can transport key enzymes involved in the APP cleavage pathway, including β‐secretase.^[^ ^26^ ^]^ Upon selecting these NEV marker/protease combinations, we designed the proof‐of‐concept study of NEV β‐secretase activity assay by utilizing CSF samples for the following reasons: i) CSF is in direct contact with the brain, as it circulates through the brain's ventricles, resulting in a high concentration of NEVs, ii) CSF is isolated from other body fluids, minimizing the presence of EVs originating from non‐neuronal sources, and iii) CSF contains significantly lower levels of soluble proteins compared to other biofluids. These characteristics make CSF an ideal model biofluid for this study, offering a relatively pure environment with a predominance of NEVs.

To meet the standardized guidelines for EV characterization outlined in MISEV2023,^[^ ^29^ ^]^ we performed comprehensive analyses of CSF‐derived NEVs using various techniques, including NTA, TEM, and SEM. These analyses confirmed the NEVs’ size (171.5 ± 59 nm), concentration (3.19 × 10^9^ NEVs mL^−1^), and morphology. Additionally, TEM imaging with immunogold staining verified the colocalization of NEV markers with β‐secretase on the enriched NEVs. Following successful characterization, we evaluated the assay's ability to differentiate AD patients from HCs by testing CSF samples from a study cohort of 15 AD patients and 15 HCs, achieving AUROCs of 0.956 and 0.969 for L1CAM(+)‐NEVs and NCAM(+)‐NEVs, respectively. It is worth noting that outliers from AD patient group observed in the study were likely due to the use of postmortem CSF samples with inconsistent postmortem intervals, which could have affected the integrity of NEVs in the samples. Despite this, the proof‐of‐concept study using clinical CSF samples successfully demonstrated the assay's ability to differentiate AD patients from HCs by assessing β‐secretase activity of NEVs.

Our successful proof‐of‐concept study enabled us to transition from CSF to synthetic plasma samples for further validation of the assay's ability to quantify β‐secretase activity of NEVs in plasma, a more accessible biofluid compared to CSF. This quantitative capability in plasma condition underpins the assay's potential for detecting early onset of ADNPC in a noninvasive manner. By spiking varying concentrations of NEVs from the CSF of AD patient into EV‐depleted HC plasma, we established a strong linear correlation for quantifying β‐secretase activity across a broad NEV concentration range of 5 × 10^7^–10^9^ NEVs mL^−1^. This validated the assay's capability for quantitative assessment of NEV‐associated β‐secretase even in plasma sample.

Following this validation, we proceeded with a retrospective case‐control study by applying the assay to the clinical plasma samples from a validation cohort of 55 AD patients and 55 HCs. The assay demonstrated excellent diagnostic performance, with AUROCs of 0.968 and 0.954 for L1CAM(+)‐NEVs and NCAM(+)‐NEVs, respectively, in distinguishing AD patients from HCs. When we independently evaluated the diagnostic accuracy for age‐matching and non‐age‐matching HCs, a slight reduction in discrimination power was observed in the age‐matching group. This was attributed to slightly elevated β‐secretase activity in age‐matching HCs compared to non‐age‐matching group, suggesting that β‐secretase activity may increase in older individuals at higher risk, even before the onset of ADNPC. To further enhance diagnostic accuracy, we employed a logistic regression model to combine readouts from both NEV subpopulations, generating the NEV β‐secretase Activity Score. This score achieved exceptional accuracy, with an AUROC of 0.986, demonstrating the assay's high sensitivity (96%) and specificity (93%). Importantly, the NEV β‐secretase Activity Score showed a strong correlation with AD patients’ MMSE scores, a standardized measure of cognitive performance.

Our assay achieves its significant diagnostic performance by leveraging the unique properties of NEVs, which preserve enzyme integrity and enhance signal specificity. As summarized in Table S4 (Supporting Information), existing methods employing β‐secretase as an AD biomarker have exhibited suboptimal diagnostic performance. These methods can be categorized into two groups: **Group 1**) detection of β‐secretase levels and **Group 2**) detection of β‐secretase activity. Within **Group 1**, studies have focused on quantifying β‐secretase lncRNA (BACE1‐AS) levels (**Group 1.1**) or β‐secretase protein expression levels (**Group 1.2**) in CSF, plasma, or whole blood. However, these quantification‐based approaches have shown limited diagnostic performance, with AUROC values ranging from 0.55 to 0.72. To address these limitations, **Group 2** studies have attempted to directly assess β‐secretase activity in serum and CSF. Despite this shift in approach, these assays still exhibit modest diagnostic performance (AUROC < 0.8), likely due to enzymatic degradation and non‐neuronal β‐secretase sources diluting disease‐specific signals. Our approach overcomes these challenges by enriching NEVs followed by assessing β‐secretase activity within the enriched NEVs, ensuring a more reliable signal from intact β‐secretase and minimizing background interference from non‐neuronal sources. As a result, our assay achieves a significantly higher diagnostic performance compared to non‐EV‐based β‐secretase detection techniques, highlighting its novelty and superior diagnostic potential in AD diagnostics.

Given its diagnostic performance in the retrospective case‐control study, the NEV β‐secretase activity assay could have strong potential as a complementary tool to PET scans and CSF tests for earlier detection of ADNPC followed by guidance of timely treatment interventions for AD. This potential stems from several distinct advantages: i) its high specificity, achieved through a double‐layered workflow that requires colocalization of NEV markers (i.e., L1CAM and NCAM) with AD‐associated protease (i.e., β‐secretase) on NEVs to produce positive readout; ii) its high sensitivity, which is ensured by click chemistry‐mediated enrichment of NEVs, followed by signal amplification from enzymatic cleavage of β‐secretase FRET probes; iii) its targeting of upstream β‐secretase activity rather than downstream amyloid‐β products, facilitating earlier detection of ADNPC; iv) its potential for noninvasive diagnosis of AD by assessing β‐secretase activity in NEVs derived from blood samples; and v) its readout compatibility with widely available fluorescence plate readers, ensuring easy use in various laboratory settings.

Despite the promising performance of the NEV β‐secretase activity assay, several limitations must be acknowledged. First, the proof‐of‐concept and retrospective case‐control studies using clinical CSF and plasma samples from relatively small cohorts limit the statistical power to fully demonstrate the assay's diagnostic power in a clinical setting. Larger‐scale studies are necessary to address this limitation. Second, our current study focused on the biomarker development to establish a solid foundation for its diagnostic potential by distinguishing AD patients from HCs. However, this study does not fully demonstrate the assay's capability for detecting earlier stages of ADNPC. To address this limitation, future studies will expand the cohort to include AD patients at earlier stages such as mild cognitive impairment, as well as cognitively normal older adults with amyloid‐β plaques, enabling a more thorough evaluation of the assay's utility for early ADNPC diagnosis. Third, further analytical validation is required by correlating the assay's readouts with PET scans and CSF tests, which are two contemporary gold standards for detecting AD, to comprehensively confirm the assay's diagnostic performance. Fourth, longitudinal studies are required to validate the assay's capability for monitoring disease progression and treatment effects. Fifth, it is crucial to assess the assay's accuracy in distinguishing AD from other non‐AD dementias, such as frontotemporal dementia and amyotrophic lateral sclerosis, to ensure the development of a highly specific screening method for AD. Lastly, since AD is a multifactorial neurodegenerative disorder characterized by the interplay of various pathological mechanisms, incorporating other enzymes involved in AD pathogenesis should be considered to provide a more comprehensive insight for early ADNPC detection. For instance, increasing evidence suggests that elevated levels of matrix metalloproteinases (MMPs), including MMP2, MMP9, and MMP14, in the brains of AD patients contribute to pathophysiological processes such as neuroinflammation.^[^ ^37^ ^]^ Expanding the capacity of this assay to include these factors could lead to a more thorough evaluation of AD and offer better insights for personalized care and treatment strategies.

## 4 Conclusion

In this study, we successfully developed and validated the NEV β‐secretase activity assay, a noninvasive method for detecting the early onset of ADNPC. This assay assesses β‐secretase activity of NEVs enriched by one of the two NEV markers (i.e., L1CAM or NCAM), generating a distinctive β‐secretase activity profile for each patient. In a proof‐of‐concept demonstration with CSF samples from a study cohort, the NEV β‐secretase activity assay accurately differentiated AD patients from HCs. When subsequently applied to plasma samples from a validation cohort, the assay also exhibited remarkable performance in distinguishing AD patients from HCs, achieving an AUROC of 0.968 for L1CAM(+)‐NEVs and 0.954 for NCAM(+)‐NEVs. Additionally, the NEV β‐secretase Activity Score, a logistic regression model generated by combining the readouts from two subpopulations of NEVs, achieved more enhanced detection accuracy (AUROC = 0.986), with high sensitivity (96%) and specificity (93%). Notably, this score showed a strong correlation with AD patients’ MMSE scores. The NEV β‐secretase activity assay represents a significant advancement in leveraging the diagnostic potential of NEVs, enabling noninvasive, quantitative, and reproducible assessment of β‐secretase activity. This study marks a pivotal evolution of our click chemistry‐mediated EV enrichment technology, as we integrated FRET probes for downstream readouts. Ultimately, this noninvasive functional assay for detecting the early onset of ADNPC can open new avenues for the general noninvasive assessment of biochemical functions, specifically enzymatic activities, that enable us to advance beyond the simple quantification of disease‐associated proteins and mRNAs.

## 5 Experimental Section

### Instrumentation

Nanoparticle tracking analysis (NTA) was conducted by ViewSizer 3000 (HORIBA Scientific, Japan). Zeta potential was measured on the Zetasizer Nano instrument (Malvern Instruments Ltd., UK). Fluorescence readouts were measured by the microplate reader (CLARIOStar, BMG Labtech, Germany).

### Clinical CSF and Plasma Samples

CSF (15) samples from HCs were purchased from Medix Biochemica (St. Louis, MO, US) and stored at −80 °C without further processing. Additionally, 15 postmortem CSF samples were collected by following protocols of the Department of Pathology and Laboratory Medicine at UCLA. Specifically, the postmortem CSF was obtained during autopsy by inserting a needle attached to a 20 mL syringe into the lateral ventricles; 1 mL aliquots were then stored in 1.5 mL cryotubes at −80 °C until use. The clinical characteristics of these participants are provided in Table S2 (Supporting Information). Furthermore, 110 plasma samples (55 from HCs and 55 from AD patients) were purchased from ProteoGenex (Inglewood, CA, US) and stored at −80 °C without further processing. The clinical characteristics of these participants are summarized in Table S3 (Supporting Information).

### Collection of NEVs from the CSF of AD Patient

NEVs were isolated from the CSF of AD patient using ExoQuick (System Biosciences, US) following the manufacturer's instruction. Specifically, CSF was centrifuged at 3,000 g for 15 min at 4 °C to remove cellular debris. Then, 0.25 mL of CSF supernatant was mixed with 63 µL of ExoQuick solution, followed by incubation at 4 °C for 30 min. The mixture was centrifuged at 1,500 g for 30 min at 4 °C, and the supernatant was carefully aspirated not to disturb the precipitated NEVs in pellet. The pellet was resuspended in 0.25 mL of 0.22 µm filtered PBS, which was immediately used to characterize NEVs.

### Characterization of NEVs’ Size Distribution and Concentration

The size distribution and concentration of NEVs were determined by NTA. NEV samples were diluted into 1 mL of 0.22 µm filtered PBS at dilution rates ranging from 100 to 10,000‐fold. Each sample was analyzed in three replicates.

### SEM Imaging

Scanning electron microscopy (SEM) images were obtained using a Supra 40VP SEM (Zeiss, Germany) at an accelerating voltage of 20 kV. NEV samples and NEV‐immobilized Click MagBeads were cast onto a silicon wafer and air‐dried at room temperature overnight. The dried samples were sputter‐coated with a gold target (Ted Pella, US).

### TEM Imaging

Transmission electron microscopy (TEM) images were acquired using a Tecnai 12 Quick Cryo‐EM (FEI, US). Prior to sample preparation, a 400‐mesh carbon‐coated copper grid (Ted Pella, US) was glow discharged using the PELCO easiGlow Glow Discharge Cleaning System (Ted Pella, US) to facilitate hydrophilization, and the grid was used within 60 min. NEV samples and NEV‐immobilized Click MagBeads were applied to the glow discharged grid and incubated for 2 min at room temperature. Excess samples were removed by blotting with filter paper, and the grid was washed three times by 5% uranyl acetate. Subsequently, the samples were stained with 5% uranyl acetate for 1 min.

### Synthesis of Click MagBeads

Amine‐grafted Dynabeads M‐270 (200 µL, Thermo Fisher Scientific, US) were washed twice with dry dimethyl sulfoxide (DMSO). The beads were then resuspended in dry DMSO (50 µL) containing triethylamine (90 µmol) and reacted with mTz‐PEG4‐NHS (0.9 µmol) for 60 min. Then, mPEG4‐NHS (18 µmol in dry DMSO) was added, and the mixture was incubated for additional 60 min. Residual NHS was quenched by adding 120 µL of Tris buffer (pH 8.4) and reacting for 10 min. The beads were washed three times with distilled water (DW) and twice with ethanol (EtOH), then dried at room temperature. The dry beads were stored at 4 °C with protection from light and reconstituted in PBST (0.05%) before use.

### Characterization of Click MagBeads

The zeta potential was measured at each step of surface modification of Click MagBeads. The concentration of the beads was maintained at 0.1 mg mL^−1^ in PBS, and each sample was replicated over three runs. The amount of mTz on Click MagBeads was determined by back titration of TCO‐Cy5 after incubation with the beads. A standard curve of TCO‐Cy5 was generated in the range of 0–5 µM in PBS using fluorescence at λ_ex_ = 620 nm and λ_em_ = 680 nm. 0.5 mg of Click MagBeads was dispersed in 200 µL of 5 µM TCO‐Cy5 solution and allowed to react for 60 min at room temperature. The Cy5‐labeled Click MagBeads were magnetically separated, and the Cy5 fluorescence of the supernatant was measured to determine the reacted amount of TCO‐Cy5. The Cy5‐labeled Click MagBeads were visualized using fluorescence microscopy (Nikon 90i, Nikon, Japan) at far red channel.

### Synthesis of TCO‐Grafted Antibodies

Anti‐L1CAM (R&D Systems, US) or anti‐NCAM (Abcam, UK) antibody (1 mg mL^−1^, 20 µL) and TCO‐PEG4‐NHS (1 mM, 2.7 µL, Click Chemistry Tools) were mixed in net volume 100 µL of PBS (pH 8.5) and incubated for 60 min at room temperature with shaking. Unconjugated TCO‐PEG4‐NHS was removed by Zeba 40 kDa column (Thermo Fisher Scientific, US) as per manufacturer's instruction. The obtained amount of TCO‐grafted antibodies was determined by spectrophotometer (NanoDrop 2000, Thermo Fisher Scientific, US) with absorbance at wavelength of 280 nm. The degree of labeling (DOL) of TCO was calculated by methyltetrazine‐Cy5 (mTz‐Cy5) labeling method. In details, TCO‐grafted antibodies were reacted with excess amount of mTz‐Cy5 for 60 min at room temperature and purified with Zeba 40 kDa column. DOL of Cy5 was determined using the protein Cy5 labeling mode of NanoDrop, yielding an average of ∼3 TCO per antibody across the entire batch, regardless of antibody type.

### Click Chemistry‐Mediated NEV Enrichment from CSF and Plasma

NEVs in both CSF and plasma were enriched using Click MagBeads based on the optimized condition from the previous study. In brief, 100 ng of one of the two TCO‐grafted antibodies (i.e., TCO‐anti‐L1CAM or TCO‐anti‐NCAM) in 0.4 mL of PBS were mixed with 0.4 mL of CSF or plasma for 45 min at room temperature with shaking. The CSF or plasma containing TCO‐labeled NEVs was incubated with 100 µg of Click MagBeads, which were preblocked in 100 µL of 10% BSA, for 45 min at room temperature with shaking, followed by a magnetic separation for 30 s. The NEV‐enriched Click MagBeads were washed three times by PBST (0.05%) before analyses.

### Characterization of NEV‐Enriched Click MagBeads

NEV‐enriched Click MagBeads were analyzed by SEM and immunogold TEM. NEVs in the CSF of AD patient were enriched onto Click MagBeads with TCO‐anti‐L1CAM and TCO‐anti‐NCAM using the protocol described in “Click Chemistry‐Mediated NEV Enrichment from CSF and Plasma” section. The NEV‐enriched Click MagBeads were fixed in 4% paraformaldehyde (PFA) solution for 30 min at room temperature. For SEM specimens, the samples were washed with ddH_2_O and prepared as described in “SEM Imaging” section. For immunogold staining, the fixed NEV‐enriched Click MagBeads were incubated with either anti‐CD63 (MyBioSource, goat, 1:50 dilution) or anti‐β‐secretase (R&D Systems, goat, 1:50 dilution) in 1% BSA for 60 min at room temperature and washed twice with PBS. Subsequently, the sample was incubated with anti‐goat IgG nanogold (Jackson ImmunoResearch, 18 nm, 1:20 dilution) in 1% BSA for 30 min. The nanogold‐labeled sample was dropped onto glow discharged TEM grids, and the specimen was prepared as described in “TEM Imaging” section without staining.

### Investigation of β‐Secretase Enzymatic Kinetics

The progress curve of β‐secretase was monitored by using microplate reader. 0.3 µL of β‐secretase FRET probes (Sigma‐Aldrich, US) at varying concentrations in DMSO were added to 24.7 µL recombinant human β‐secretase (Sigma‐Aldrich, US) solution (20 mM sodium acetate at pH 4.5, 50 mM NaCl, 0.05% Triton X‐100, and 160 nM recombinant human β‐secretase in final concentration), followed by enzyme reaction at 37 °C for 60 min with fluorescence signal measurement (λ_ex_ = 350 nm and λ_em_ = 490 nm) at every 5 min. The hydrolysis rates of β‐secretase FRET probes at various concentrations obtained from the progress curve were fitted to the Michaelis‐Menten equation to determine the kinetic parameters of β‐secretase (V_max_, K_M_, k_cat_, and k_cat_/K_M_). To obtain the dynamic range, the final concentration of β‐secretase FRET probes was fixed at 12 µM, approximately three times the K_M_, and the probe solution was added in serial dilutions of human recombinant β‐secretase solution. For FRET probe specificity test, 160 nM of enzymes (β‐secretase, α‐secretase, MMP14, and MMP2) were incubated with 12 µM of β‐secretase FRET probes at 37 °C.

### Preparation of Synthetic Plasma

Synthetic plasma samples mimicking the plasma of AD patients were prepared to validate the assay's reliability for detecting NEVs in plasma condition. We first prepared EV‐depleted HC plasma through ultracentrifugation. In brief, HC plasma was transferred to six Ultra‐Clear Tubes (38 mL per tube, Beckman Coulter, Inc., US) and then ultracentrifuged at 100,000 g for 90 min at 4 °C. The top two‐thirds (approximately 26 mL per tube) of plasma was carefully collected as EV‐depleted HC plasma. The synthetic plasma samples were then prepared by spiking the CSF of AD patient into EV‐depleted HC plasma, adjusting the final NEV concentration to the desired level. The mixture was aliquoted into duplicates of 0.4 mL each for further analysis.

### NEV β‐Secretase Activity Assay Using CSF and Plasma

NEVs in both CSF and plasma were enriched using Click MagBeads following the protocol described in “Click Chemistry‐Mediated NEV Enrichment from CSF and Plasma” section. 10 µL of lysis buffer (26 mM sodium acetate at pH 4.5, 125 mM NaCl, and 0.1% Triton X‐100) was added to lyse the NEVs enriched onto Click MagBeads, followed by incubation at room temperature for 30 min with shaking. The Click MagBeads were separated by magnetic separation for 30 s and the supernatant was transferred to 384‐well plate (Corning, US). 15 µL of β‐secretase FRET probe solution (16 mM sodium acetate at pH 4.5 and 20 µM β‐secretase FRET probes) was added and time‐dependent fluorescence readouts were measured using microplate reader with identical measurement setting described in “Investigation of β‐Secretase Enzymatic Kinetics” section. The β‐secretase activity was determined by calculating the hydrolysis rate (RFU s^−1^) from the linear range within the time‐dependent fluorescence readout curve.

### ELISA‐based Quantification of β‐secretase Expression Levels Within the Enriched NEVs

NEVs in plasma were enriched using Click MagBeads following the protocol described in Click Chemistry‐Mediated NEV Enrichment from CSF and Plasma section. Then, the β‐secretase expression levels were quantified using Human Beta‐Secretase 1 ELISA Kit (Invitrogen, US), by following the manufacturer's instruction. Briefly, 100 µL of lysis buffer (0.1% Triton X‐100 in 1X Assay Diluent) was added to lyse the NEVs captured on Click MagBeads, followed by incubation at room temperature for 30 min with shaking. The lysate was transferred to an ELISA plate and incubated at room temperature for 2.5 h with gentle shaking. After discarding the solution, the wells were washed four times with 300 µL of 1X Wash Buffer. Next, 100 µL of biotinylated antibody was added, and the plate was incubated for 1 h at room temperature with gentle shaking. Following four additional washes, 100 µL of Streptavidin‐HRP was added and incubated for 45 min under the same conditions. The wells were washed again before adding 100 µL of TMB Substrate and incubating for 30 min. The reaction was stopped by adding 50 µL of Stop Solution, and absorbance was measured at 450 nm using a microplate reader (CLARIOStar, BMG Labtech, Germany).

### Statistical Analysis

OriginLab 2019 (OriginLab Corp. US) or MedCalc (MedCalc Software Ltd., Belgium) was used for statistical analyses. For two‐group comparisons, a one‐way ANOVA was used. For all statistical tests, *p*‐values less than 0.05 were considered significant. The results were reported as mean ± standard deviation. Details of sample size were included in figure legends. The optimal cutoff was calculated to maximize the sensitivity and specificity for ROC analysis. The weight values for each variable in the logistic regression model were determined through the logistic regression algorithm, which optimizes their contributions to maximize classification accuracy.

## Conflict of Interest

H.‐R.T. would like to disclose that he has financial interests in CytoLumina Technologies Corp., Pulsar Therapeutics Corp., and Eximius Diagnostics Corp. Y.Z. is a co‐founder and shareholder in Eximius Diagnostics Corp. All other authors declare they have no competing interests.

## Author Contributions

H.K., J.L., Y.Z., and H.‐R.T. conceptualized and designed the study. H.K. performed the experiments. J.L., H.‐Y.C., and M.D.S. prepared and characterized the Click MagBeads. J.L. characterized the NEVs. H.K., J.L., Y.‐R.J., R.Z., Y.Z., and H.‐R.T. analyzed the data. R.Z. prepared and characterized the antibodies. H.K., J.L., A.Q., Y.Z., and H.‐R.T. wrote the manuscript and created the figures, with input from all authors. Q.H. revised the figures. C.K.W., T.Z., H.V.V., and S.M. collected the postmortem CSF samples. H.K., Y.X., L.G., M.C.M., E.M.P., Z.S.T., K.V., Y.Z., H.‐R.T. contributed to evaluating the assay's diagnostic accuracy through statistical analyses and comparisons with existing AD detection methods.

## Supporting information



Supporting Information

## Data Availability

The data that support the findings of this study are available from the corresponding author upon reasonable request.
