# Supplementary material for: Noninvasive Assessment of β‐Secretase Activity Through Click Chemistry‐Mediated Enrichment of Neuronal Extracellular Vesicles to Detect Alzheimer's Disease
Source: Adv Sci (Weinh). 2025 Apr 17;12(26):2415289. doi: 10.1002/advs.202415289 (PMC12245089; doi:10.1002/advs.202415289)
Supplement: Supplementary file 1 — Supporting Information [file ADVS-12-2415289-s001.docx]

Supporting Information

Noninvasive Assessment of β-secretase Activity Through Click Chemistry-Mediated Enrichment of Neuronal Extracellular Vesicles to Detect Alzheimer’s Disease

Hyoyong Kim, Junseok Lee*, Audrey Qian, You-Ren Ji, Ryan Zhang, Qixin Hu, Christopher Kazu Williams, Han-Yu Chuang, Matthew D. Smalley, Yaya Xu, Liang Gao, Mary C. Mayo, Ting Zhang, Edwin M. Posadas, Zaldy S. Tan, Harry V. Vinters, Keith Vossel, Shino Magaki, Yazhen Zhu*, and Hsian-Rong Tseng*


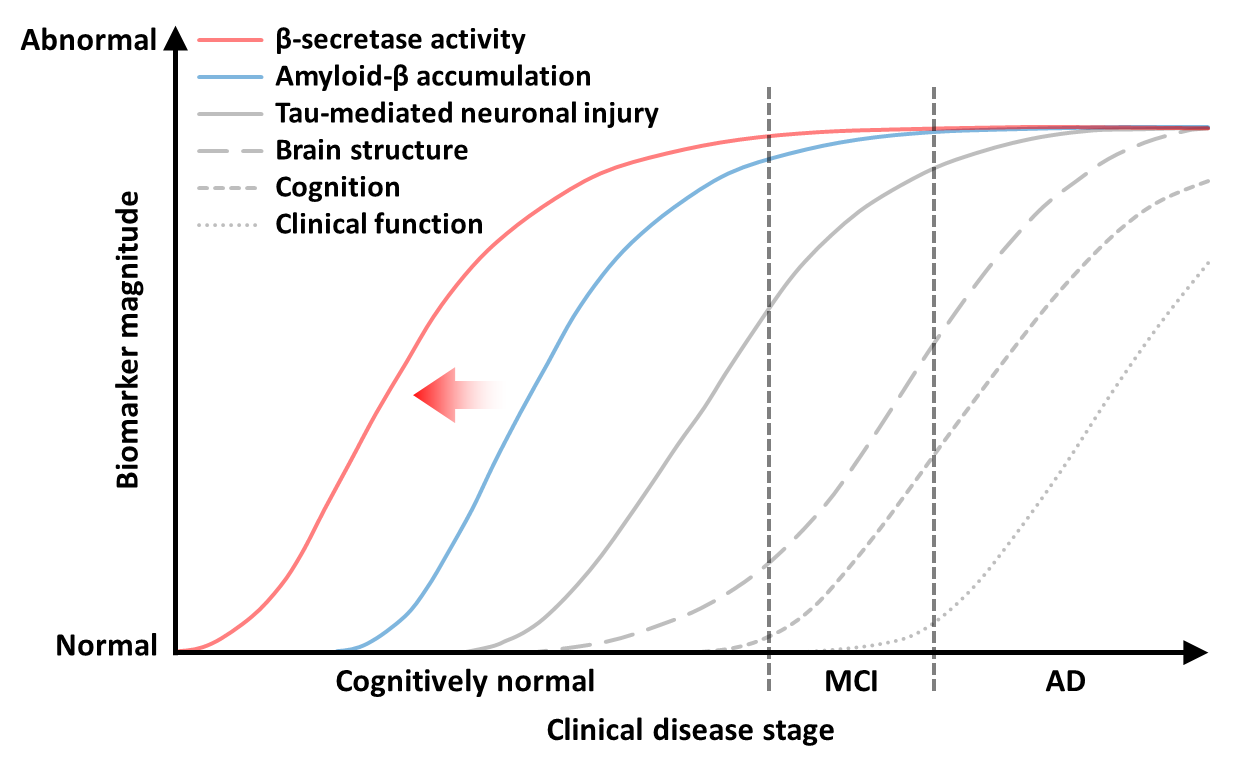


**Figure S1.** Diagram illustrating the severity of biomarker abnormalities across different stages of AD progression.^[1]^ Existing methods identify amyloid-β accumulation, an early indicator of ADNPC compared to different biomarkers, by detecting changes in amyloid-β peptides in CSF or blood or by identifying amyloid-β plaques through PET scans. However, these methods can only diagnose the disease once the plaques have formed and reached detectable levels. To overcome this limitation, a promising alternative is to assess upstream β-secretase activity, potentially enabling the detection of ADNPC at an even earlier stage than current approaches.


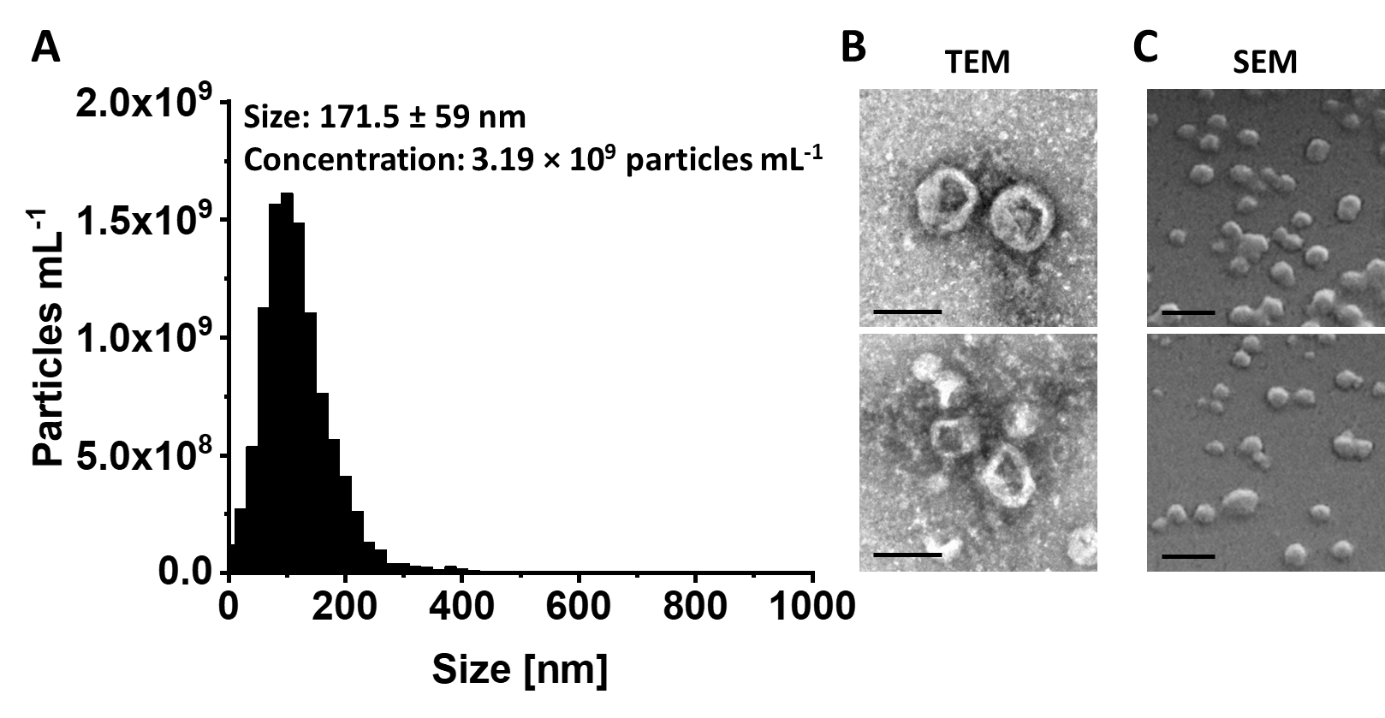


**Figure S2.** Characterization of NEVs in the CSF of AD patient. NEVs isolated from the CSF of AD patient were characterized by A) NTA to measure their size and concentration, and B) TEM and C) SEM (scale bar = 200 nm) to confirm the morphology and integrity of NEVs.


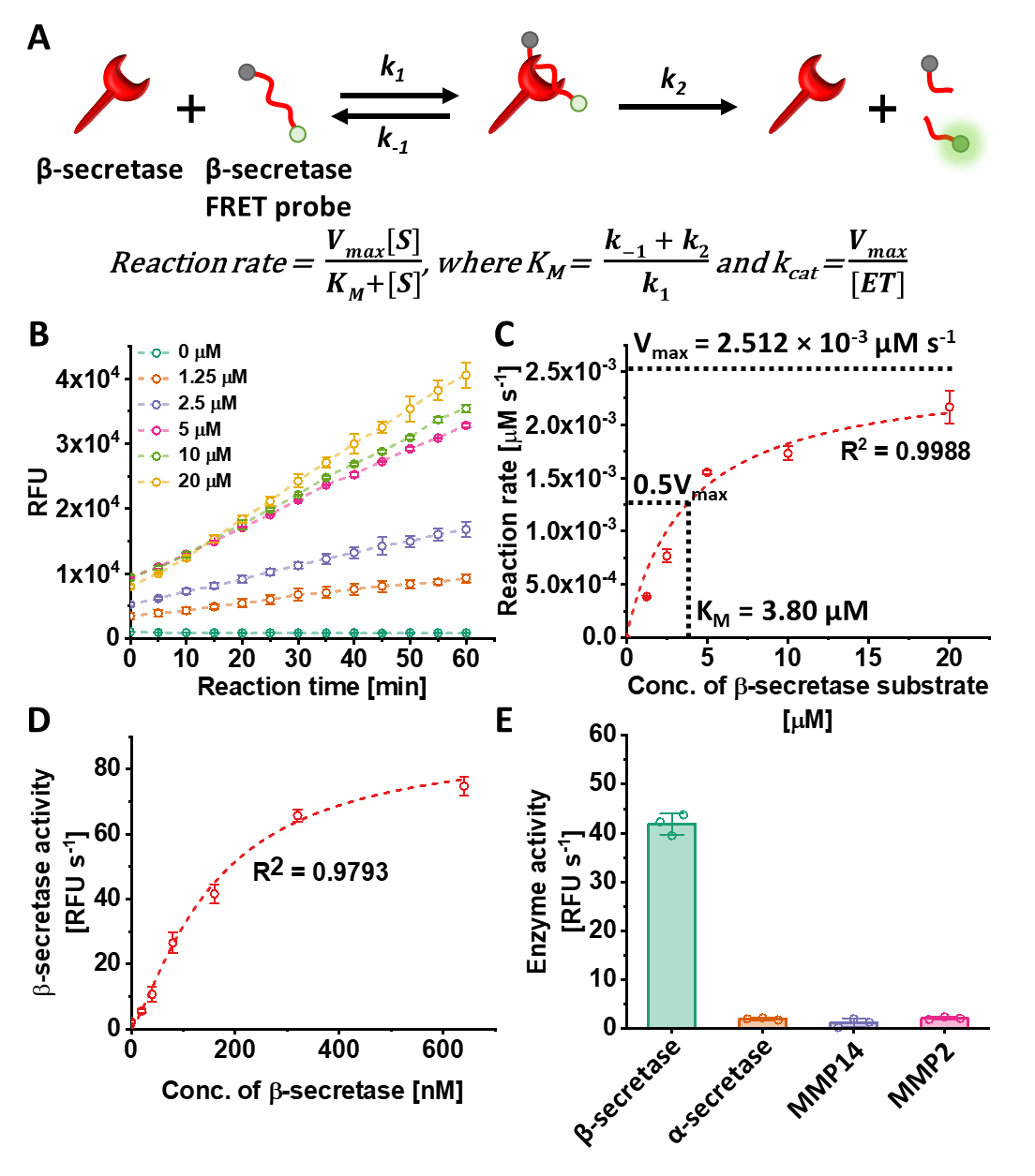


**Figure S3.** Investigation of β-secretase enzymatic kinetics. A) Schematic illustration of the β-secretase activity assay using recombinant human β-secretase. B) Time-dependent measurement of fluorescence generated by the hydrolysis of the β-secretase FRET probes by recombinant human β-secretase and C) corresponding Michaelis-Menten plot for determining the kinetic parameters (V_max_ = 2.512 × 10^-3^ μM s^-1^, K_M_ = 3.80 μM, k_cat_ = 0.0157 s^-1^, and k_cat_/K_M_ = 4,130.98 s^-1^ M^-1^). D) Dynamic range of the β-secretase activity assay. E) Verification of the specificity of the β-secretase FRET probes.


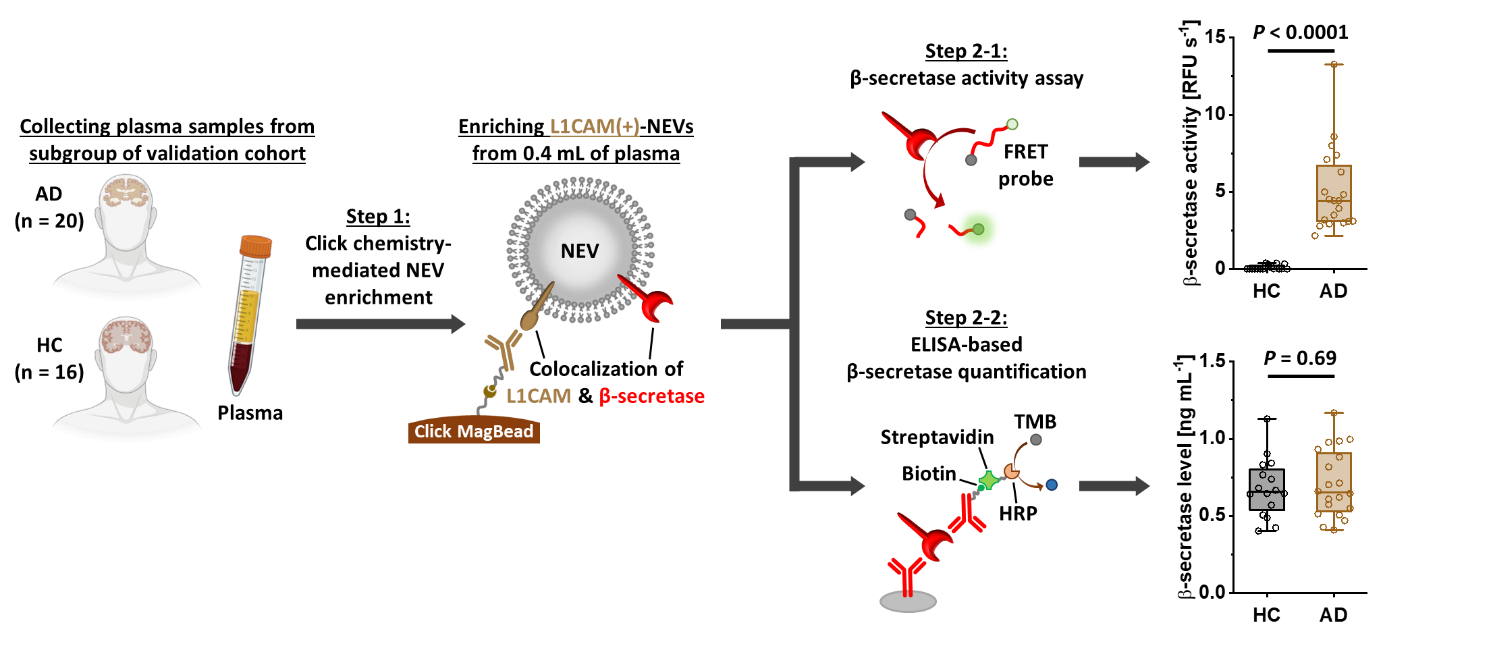


**Figure S4.** Comparison of enzymatic activity and expression levels of β-secretase in L1CAM(+)-NEVs from AD patients (n = 20) and HCs (n = 16).


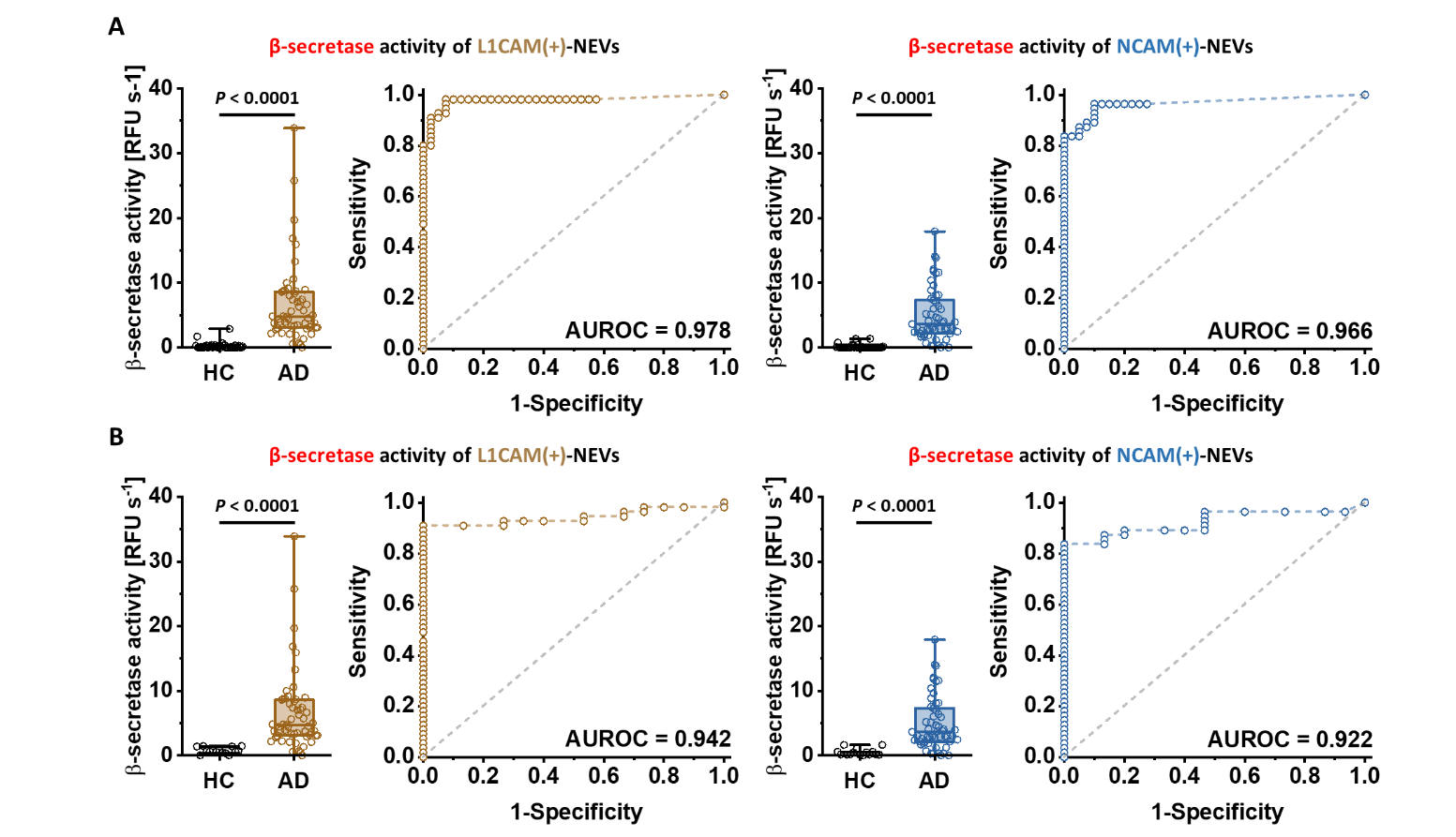


**Figure S5.** Box charts and corresponding ROC curves of the two subpopulations of NEVs for differentiating AD patients from A) non-age-matching and B) age-matching HCs.

**Figure S6.** Correlation between ages of AD patients and respective NEV β-secretase Activity Scores.

**Table S1.** Summary of literature survey for identifying markers for enriching NEVs.

| **Biofluid** | **NEV marker for enriching NEVs** | **Reference** |
| --- | --- | --- |
| Plasma, Serum | L1CAM, NCAM | [2] |
| - | L1CAM, NCAM, CSPG4/PDGFR | [3] |
| - | L1CAM, NCAM | [4] |
| CSF | NCAM, MAPT, NPTX2, SCG2, VGF, GFAP, SST | [5] |
| - | L1CAM, NCAM, IB4, Annexin IV/V, CD81, CD82 | [6] |
| Blood | L1CAM, NCAM | [7] |
| Plasma | NCAM | [8] |
| Plasma | L1CAM, NCAM | [9] |
| Plasma | NCAM, ABCA1 | [10] |
| Plasma | NCAM, Amphiphysin 1 | [11] |

**Table S2.** Clinical characteristics of HCs and AD patients for proof-of-concept demonstration of the NEV β-secretase Activity Assay using clinical CSF samples.

| **Characteristics** | **Disease stage** | | **Total** |
| --- | --- | --- | --- |
|  | **HCs** | **AD patients** |  |
| No. of subjects | 15 | 15 | 30 |
| Age, years |  |  |  |
| Mean (SD) | 71.2 (8.5) | 80.2 (8.9) | 75.7 (10.0) |
| Median | 71 | 81 | 76 |
| IQR | 67–76 | 73–87 | 70–83 |
| Sex, No. (%) |  |  |  |
| Male | 8 (53.3) | 9 (60) | 17 (56.7) |
| Female | 7 (46.7) | 6 (40) | 13 (43.3) |

**Table S3.** Clinical characteristics of HCs and AD patients for evaluation of the NEV β-secretase Activity Assay using clinical plasma samples.

| **Characteristics** | **Disease stage** | | | **Total** |
| --- | --- | --- | --- | --- |
|  | **Non-age-matching HCs** | **Age-matching HCs** | **AD patients** |  |
| No. of subjects | 40 | 15 | 55 | 110 |
| Age, years |  |  |  |  |
| Mean (SD) | 63.9 (2.1) | 76.7 (6.8) | 78.4 (10.1) | 72.9 (10.3) |
| Median | 63.5 | 73 | 77 | 71 |
| IQR | 62–65 | 72–83 | 71–87 | 64–83 |
| Sex, No. (%) |  |  |  |  |
| Male | 29 (72.5) | 10 (66.7) | 24 (43.6) | 63 (57.3) |
| Female | 11 (27.5) | 5 (33.3) | 31 (56.4) | 47 (42.7) |
| MMSE |  |  |  |  |
| Mean (SD) | - | - | 14.7 (4.1) |  |
| Median | - | - | 16 |  |
| IQR | - | - | 12–17 |  |

**Table S4.** Summary of β-secretase detection methods for AD diagnosis.

| **Sample** | **Target** | **Detection method** | **Study cohort** | **Performance for differentiating AD from HC** | | | | **Ref** |
| --- | --- | --- | --- | --- | --- | --- | --- | --- |
|  |  |  |  | ***P* value** | **Sensitivity** | **Specificity** | **AUROC** |  |
| **This work (NEV β-secretase Activity Assay)** | | | | | | | | |
| Plasma | β-secretase  Activity | FRET | HC: 55  AD: 55 | < 0.0001 | 0.96 | 0.93 | 0.986 | - |
| **Group 1.1 – Detection of β-secretase lncRNA level** | | | | | | | | |
| Plasma | β-secretase  LncRNA | RT-qPCR | HC: 72  AD: 88 | 0.006 | - | - | 0.67 | [12] |
| **Group 1.2 – Detection of β-secretase expression level** | | | | | | | | |
| Blood | β-secretase  level  (in platelets & leukocytes) | Western  blot | HC: 20  AD: 20 | - | Platelets: 0.78  Leukocytes: 0.78 | Platelets: 0.67  Leukocytes: 0.67 | Platelets: 0.67  Leukocytes: 0.72 | [13] |
| Plasma | β-secretase  level | ELISA | HC: 20  AD: 21 | 0.02 | 0.76 | 0.65 | 0.71 | [14] |
| CSF | β-secretase  level | ELISA | HC: 20  AD: 50 | 0.487 | - | - | 0.55 | [15] |
| **Group 2 – Detection of β-secretase activity** | | | | | | | | |
| Serum | β-secretase  Activity | FRET | HC: 151  AD: 115 | < 0.001 | 0.7 | 0.75 | 0.76 | [16] |
| CSF | β-secretase  Activity | FRET | HC: 21  AD: 21 | - | 0.6 | 0.86 | - | [17] |
| CSF | β-secretase  Activity | ELISA | HC: 65  AD: 75 | 0.1 | - | - | - | [18] |
| Serum | β-secretase  Activity | FRET | HC: 204  AD: 175 | < 0.001 | 0.73 | 0.7 | 0.77 | [19] |

References

[1] C. R. Jack, D. S. Knopman, W. J. Jagust, L. M. Shaw, P. S. Aisen, M. W. Weiner, R. C. Petersen, J. Q. Trojanowski, *Lancet Neurol.* **2010**, *9*, 119.

[2] M. S. Fiandaca, D. Kapogiannis, M. Mapstone, A. Boxer, E. Eitan, J. B. Schwartz, E. L. Abner, R. C. Petersen, H. J. Federoff, B. L. Miller, *Alzheimers Dement.* **2015**, *11*, 600.

[3] C. Vandendriessche, D. Kapogiannis, R. E. Vandenbroucke, *Adv. Drug Deliv. Rev.* **2022**, *190*, 114486.

[4] J. Pei, C. P. Palanisamy, S. Jayaraman, P. M. Natarajan, V. R. Umapathy, J. R. Roy, D. Thalamati, R. M. Ahalliya, G. V. Kanniappan, M. Mironescu, *Ageing Res. Rev.* **2024**, *99*, 102359.

[5] G. Sathe, C. H. Na, S. Renuse, A. K. Madugundu, M. Albert, A. Moghekar, A. Pandey, *Proteom. – Clin. Appl.* **2019**, *13*, 1800105.

[6] T. Croese, R. Furlan, *Mol. Asp. Med.* **2018**, *60*, 52.

[7] M. Shi, L. Sheng, T. Stewart, C. P. Zabetian, J. Zhang, *Prog. Neurobiol.* **2019**, *175*, 96.

[8] E. Boyer, L. Deltenre, M. Dourte, L. Colmant, E. Paître, K. Sleegers, N. Suelves, B. Hanseeuw, P. Kienlen-Campard, *Alzheimers Res. Ther.* **2024**, *16*, 141.

[9] L. Jia, Q. Qiu, H. Zhang, L. Chu, Y. Du, J. Zhang, C. Zhou, F. Liang, S. Shi, S. Wang, *Alzheimers Dement.* **2019**, *15*, 1071.

[10] Y. Li, S. Meng, W. Di, M. Xia, L. Dong, Y. Zhao, S. Ling, J. He, X. Xue, X. Chen, *CNS Neurosci. Ther.* **2022**, *28*, 1093.

[11] Y. Li, M. Xia, S. Meng, D. Wu, S. Ling, X. Chen, C. Liu, *Neurobiol. Dis.* **2022**, *171*, 105800.

[12] L. Feng, Y.-T. Liao, J.-C. He, C.-L. Xie, S.-Y. Chen, H.-H. Fan, Z.-P. Su, Z. Wang, *BMC Neurol.* **2018**, *18*, 1.

[13] J. M. d. F. Bram, L. L. Talib, H. P. G. Joaquim, T. A. Sarno, W. F. Gattaz, O. V. Forlenza, *Eur. Arch. Psychiatry Clin. Neurosci.* **2019**, *269*, 963.

[14] P. R. Manzine, M. d. S. Souza, M. R. Cominetti, *Pers. Med.* **2016**, *13*, 531.

[15] A. De Vos, H. Struyfs, D. Jacobs, E. Fransen, T. Klewansky, E. De Roeck, C. Robberecht, C. Van Broeckhoven, C. Duyckaerts, S. Engelborghs, *J. Alzheimers Dis.* **2016**, *53*, 1523.

[16] C. Cervellati, A. Trentini, V. Rosta, A. Passaro, C. Bosi, J. M. Sanz, S. Bonazzi, S. Pacifico, D. Seripa, G. Valacchi, *GeroScience* **2020**, *42*, 159.

[17] R. Holsinger, J. Lee, A. Boyd, C. Masters, S. Collins, *Neurology* **2006**, *67*, 710.

[18] C. Rosén, U. Andreasson, N. Mattsson, J. Marcusson, L. Minthon, N. Andreasen, K. Blennow, H. Zetterberg, *Neuromol. Med.* **2012**, *14*, 65.

[19] G. Zuliani, A. Trentini, V. Rosta, R. Guerrini, S. Pacifico, S. Bonazzi, A. Guiotto, A. Passaro, D. Seripa, G. Valacchi, *Sci. Rep.* **2020**, *10*, 14980.
